# The dynamic interplay between sleep and mood: an intensive longitudinal study of individuals with bipolar disorder

**DOI:** 10.1017/S0033291721005377

**Published:** 2023-06

**Authors:** K. J. S. Lewis, K. Tilling, K. Gordon-Smith, K. E. A. Saunders, A. Di Florio, L. Jones, I. Jones, M. C. O'Donovan, J. Heron

**Affiliations:** 1 Division of Psychological Medicine and Clinical Neurosciences, Cardiff University, Cardiff, UK; 2 MRC Integrative Epidemiology Unit, University of Bristol, Bristol, UK; 3 Population Health Sciences, Bristol Medical School, University of Bristol, Bristol, UK; 4 Psychological Medicine, University of Worcester, Worcester, UK; 5 Department of Psychiatry, Oxford University, Warneford Hospital, Oxford, OX3 7JX, UK; 6 Oxford Health NHS Foundation Trust, Warneford Hospital, Oxford, OX3 7JX, UK

**Keywords:** Autoregressive effect, bipolar disorder, depression, DSEM, dynamic structural equation modelling, insomnia, intensive longitudinal data, mania, Mplus, sleep

## Abstract

**Background:**

Sleep disturbances are important symptoms to monitor in people with bipolar disorder (BD) but the precise longitudinal relationships between sleep and mood remain unclear. We aimed to examine associations between stable and dynamic aspects of sleep and mood in people with BD, and assess individual differences in the strength of these associations.

**Methods:**

Participants (*N* = 649) with BD-I (*N* = 400) and BD-II (*N* = 249) provided weekly self-reports of insomnia, depression and (hypo)mania symptoms using the *True Colours* online monitoring tool for 21 months. Dynamic structural equation models were used to examine the interplay between weekly reports of insomnia and mood. The effects of clinical and demographic characteristics on associations were also assessed.

**Results:**

Increased variability in insomnia symptoms was associated with increased mood variability. In the sample as a whole, we found strong evidence of bidirectional relationships between insomnia and depressive symptoms but only weak support for bidirectional relationships between insomnia and (hypo)manic symptoms. We found substantial variability between participants in the strength of prospective associations between insomnia and mood, which depended on age, gender, bipolar subtype, and a history of rapid cycling.

**Conclusions:**

Our results highlight the importance of monitoring sleep in people with BD. However, researchers and clinicians investigating the association between sleep and mood should consider subgroup differences in this relationship. Advances in digital technology mean that intensive longitudinal data on sleep and mood are becoming increasingly available. Novel methods to analyse these data present an exciting opportunity for furthering our understanding of BD.

## Introduction

Sleep disturbance is increasingly recognised as important to monitor and treat in people with bipolar disorder (BD). Sleep loss, both insomnia and a reduced need for sleep, is included in the diagnostic criteria for depressive and manic episodes, respectively (American Psychiatric Association, 2013), but can also precede illness onset (Duffy, Jones, Goodday, & Bentall, 2016) and persist between episodes (Harvey, Schmidt, Scarnà, Semler, & Goodwin, 2005; St-Amand, Provencher, Bélanger, & Morin, 2013). Sleep loss is a self-reported prodromal symptom and commonly implicated trigger of mood episodes in BD (Jackson, Cavanagh, & Scott, 2003; Lewis et al., 2017), suggesting it could be a useful early warning sign for changes in mood. Robust evidence on whether sleep loss is a precursor of mood episodes has high translational potential, as sleep monitoring can be readily incorporated into digital technologies. However, studies to date have reported mixed findings (Bauer et al., 2006; Gruber et al., 2011; Leibenluft, Albert, Rosenthal, & Wehr, 1996; Perlman, Johnson, & Mellman, 2006).

There are several explanations for inconsistencies in current evidence. First, study inclusion criteria are variable, some including only participants with BD-I (Perlman et al., 2006) or those with a history of rapid cycling (Leibenluft et al., 1996). Participant characteristics such as bipolar subtype (Bauer et al., 2006; Lewis et al., 2017), gender (Lewis et al., 2017; Saunders, Fernandez-Mendoza, Kamali, Assari, & McInnis, 2015) and age (Schwarz et al., 2019) could influence the relationship between sleep and mood, and hence the findings from a given study. Second, sample sizes are typically small, likely partly due to the labour-intensive nature of collecting appropriate data. A third consideration is that the frequency and duration of data collection have varied (e.g. data collected at daily, weekly or monthly intervals, with durations ranging from 1 to 18 months [Bauer et al., 2006; Gruber et al., 2011; Leibenluft et al., 1996; Perlman et al., 2006]). Large longitudinal studies of people with BD with data collected at frequent intervals are needed.

Existing studies rarely disaggregate *between-* and *within*-*person* effects. Between-person effects capture *inter*individual differences, whereas within-person effects capture *intra*individual phenomena such as the impact that a reduction in sleep has on subsequent changes in mood. It is possible to decompose each person's data into a stable element (the mean for each specific individual) and a dynamic element (deviations within that person about their specific mean) which enables between- and within-person effects to be examined. This is crucial when between- and within-person effects may be of different magnitudes or even of opposite direction. For example, people are more likely to have a myocardial infarction during exercise (a within-person effect) but, on average, people who regularly exercise are at a lower risk of myocardial infarction than people who do not (between-person effect) (Curran & Bauer, 2011).

Given the disabling consequences of poor sleep and mood disturbance for extended periods (Mai & Buysse, 2008), it is important to identify who is more likely to experience these symptoms. In addition, when a person experiences a change in sleep, this could be an early warning of a change in mood, which is important for clinical monitoring. However, between-person differences may exist in a within-person phenomenon; changes in sleep could be a strong predictor of worsening mood for some individuals, but may have no effect, or even predict an improvement in mood in others. If this is true, identifying who is most likely to experience a change in mood, and what nature that mood change is likely to take following sleep disturbance, is potentially useful for clinical management of people with BD.

In this study, we aim to clarify the relationships between sleep disturbance and mood in individuals with BD using intensive longitudinal data (ILD) – data with 20 or more measurement occasions collected at short (i.e. daily, weekly) intervals (Collins, 2006). We investigate the following: (1) Are clinical and demographic characteristics associated with stable and dynamic aspects of sleep and mood? (2) Do changes in sleep disturbance predict changes in mood when we account for their bidirectional interplay? (3) Do baseline clinical and demographic characteristics moderate these relationships? Due to mixed evidence from the existing literature described above, and the fact we are investigating novel indices of sleep and mood, we are agnostic about the direction of effect we expect to find for these exploratory analyses.

## Methods

### Participants

Participants were enrolled in the Bipolar Disorder Research Network (BDRN), an ongoing research programme investigating the aetiology of bipolar and related affective disorders. Recruitment was throughout the United Kingdom via National Health Service (NHS) Community Mental Health Teams, media advertisements and patient support organisations. Eligible participants must be at least 18 years old, meet DSM-IV criteria for major affective disorder, and have onset of mood symptoms before age 65 years. Exclusion criteria are affective disorder only secondary to (i) alcohol/substance misuse, (ii) medical illness, (iii) organic brain disorder or (iv) medication. The study was approved by a Heath Research Authority NHS Research Ethics Committee (MREC/97/7/01) and by all participating NHS Trusts and Health Boards. All participants provided written informed consent after receiving a complete description of the study.

### Demographic and clinical data

DSM-IV diagnosis and other clinical and demographic variables were determined through a semi-structured interview – the Schedules for Clinical Assessment in Neuropsychiatry (Wing et al., 1990) – administered by trained research psychologists or psychiatrists, and by reviewing psychiatric case notes. A history of rapid cycling was defined as the occurrence of four or more episodes ((hypo)mania or depression) in a 12-month period. Inter-rater reliability was high, with mean kappa scores of 0.85 for DSM-IV diagnosis and 0.88 for a history of rapid cycling. Age was defined as age at the first week in which participants joined *True Colours* (described below). Our analyses required covariates to be dichotomous, therefore age was dichotomised into <55 *v*. ⩾55 years based on previous research (Smilowitz et al., 2020).

### Intensive longitudinal data: True Colours

From January 2015, all participants enrolled in the BDRN research programme were invited to use an online mood monitoring system, *True Colours* (Gordon-Smith et al., 2019). Participants received weekly email prompts to complete two validated self-report questionnaires: the Altman Self-Rating Mania Scale (ASRM; Altman, Hedeker, Peterson, & Davis, 1997) and the Quick Inventory of Depressive Symptomatology (QIDS; Rush et al., 2003). These measure the presence and severity of DSM-IV symptoms of high mood (hypomania/mania) and depression respectively over the preceding week. For brevity, we will hereafter refer to symptoms of high mood as ‘(hypo)manic symptoms’. The ASRM consists of 5 items with total scores ranging from 0–20. To avoid the possibility that associations between (hypo)manic symptoms and insomnia were driven by sleep items in the ASRM, we removed the ‘reduced need for sleep’ item from the ASRM. The QIDS comprises 16 items combined to give a total score ranging from 0 to 27. Insomnia items were removed from the QIDS total score prior to analysis. Insomnia symptoms were ascertained by summing the total score from the three QIDS insomnia items (difficulty getting to sleep, staying asleep, and early morning waking), resulting in a total score ranging from 0 to 9.

### True Colours data – eligibility criteria for the current study

True Colours data were downloaded on 25 September 2019, at which time 925 BDRN participants were enrolled in True Colours and met DSM-IV criteria for a best-estimate main lifetime diagnosis of BD-I or BD-II. Following selection criteria devised in line with current guidance for ILD (see online Supplement), 21 months of data for 649 participants were analysed.

### Statistical analysis

#### Dynamic structural equation modelling

We analysed True Colours ILD for insomnia and mood symptoms using Dynamic Structural Equation Modelling (DSEM; Asparouhov, Hamaker, & Muthén, 2018); a Bayesian technique using a Markov Chain Monte Carlo algorithm. The DSEM framework unites several modelling approaches such as time series analysis, confirmatory factor analysis and multilevel modelling. Unlike Maximum Likelihood, DSEM handles missing data by introducing additional random effects (model parameters) for each missing value (Asparouhov et al., 2018) however DSEM also assumes data are Missing At Random (Hamaker, Asparouhov, Brose, Schmiedek, & Muthén, 2018). Analyses were conducted using Mplus version 8.4 (Muthén & Muthén, 2017) and the *MplusAutomation* package in R (v3.4.4; Hallquist & Wiley, 2018).

**Fig. 1.**
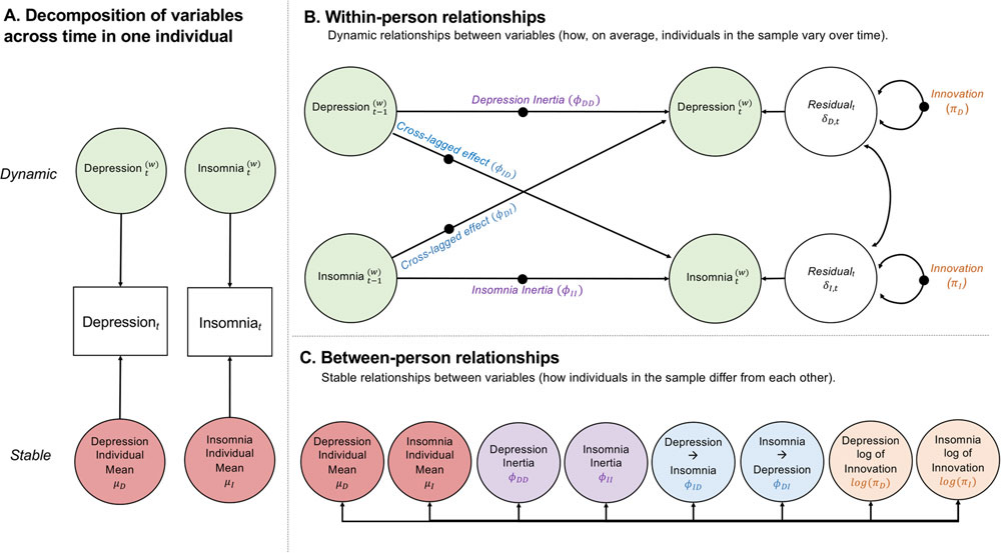
Dynamic Structural Equation Model (DSEM) of insomnia and depressive symptoms, adapted from Hamaker et al. (2018). Panel *a* shows how intensive longitudinal data can be decomposed into a dynamic (time varying) and stable (time invariant) component. Panel *b* shows relationships between time varying components of these variables (within-person relationships). The within-person effects (black circles in Panel *b*) and individual mean levels are allowed to vary across individuals, which is shown in panel *c* (between-person relationships). N.B. (i) Individual means refer to participant stable levels of depression or insomnia. (ii) The cross-lagged parameters shown in Panels B and C are not average individually standardised.

Our model, depicted in Fig. 1, can be conceptualised as a multilevel extension of a cross-lagged panel model. Using fixed and random components, the various parameters such as autoregressive effects, cross-lagged effects and residual variances can vary between individuals (Jongerling, Laurenceau, & Hamaker, 2015). We describe the key features of the model below and, where possible, relate these features to concepts traditionally studied in the field of sleep/mood research.

#### A. Decomposition of variables across time

Figure 1*a* illustrates the decomposition of the depression and insomnia data into a pair of stable and dynamic components. The stable component is different for each participant, but is assumed not to change within a participant, at least during the time window under investigation. The dynamic component, however, reflects time-specific deviations which can differ both between participants, but also within a given participant, as that person's sleep or mood fluctuates about its own stable level. Both stable and dynamic components are denoted by circles to indicate that they are latent. Therefore, rather than estimating a stable mean level for insomnia in each of the 649 participants, between-person differences in the stable component are modelled by an unmeasured normally distributed random effect with an estimated variance.

DSEM models, by default, assume no systematic change in the repeated measures over time. Consequently, the terms ‘mean’ and ‘baseline’ have both become acceptable when referring to each person's stable level. Unfortunately, each has shortcomings – ‘mean’ because any parameter which is permitted to vary also has a population mean level (the fixed effect), and ‘baseline’ because this is inextricably linked to the idea of a starting point at the first wave. For these reasons, we will use the phrase ‘individual mean’ when referring to a participant's stable level of either insomnia or mood, which frees up the terms mean and baseline for their more familiar use.

#### B. Within-person

Figure 1*b* depicts within-person relationships between insomnia and mood over time using depressive symptoms as an example. These structural relationships link the *dynamic components* of insomnia and mood, for instance, the relationship between a deviation in mood at one-time point and the deviation at the next time point.

*Inertia* (*ϕ_jj_*), also known as ‘carry-over’ or autoregression, is shown in Fig. 1*b* as arrows from measures at time *t*−1 to measures at time *t* and represents the degree to which prior (lagged) scores affect current scores (Suls, Green, & Hillis, 1998). Estimates are on a scale of −1 to 1, but usually positive (Hamaker et al., 2018) indicating that one's scores in consecutive measurements tend to be similar, particularly when the time-lags between waves are of short duration. Inertia values closer to 1 indicate that individuals take longer to return to their individual mean level after perturbations (Hamaker, 2012; Suls et al., 1998). In the context of mood, inertia might be considered a measure of regulation. For example, if two individuals experience increased depression symptoms during a given week, the individual with low inertia may exhibit reduced symptoms by the following week, but the person with high inertia may remain depressed for longer. In our tables and figures, inertia estimates are denoted by *ϕ*_ΙΙ_ for insomnia, *ϕ*_DD_ for depressive symptoms and *ϕ*_MM_ for (hypo)mania symptoms.

**Fig. 2.**
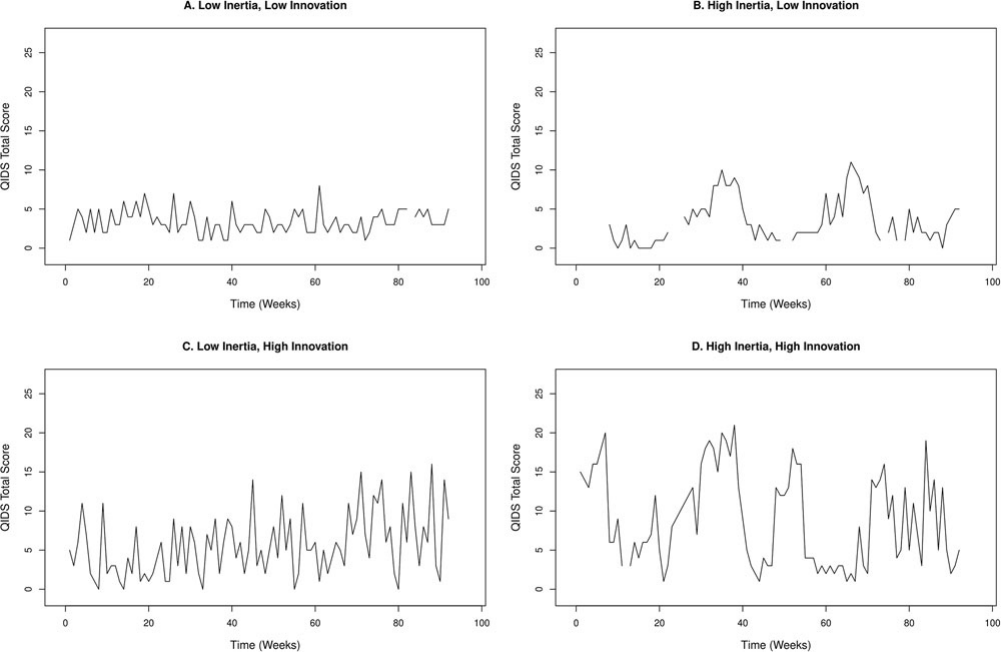
Examples of time series data from participants with high and low levels of inertia (*ϕ_ii_*) and innovation (log(*π_j_*)) in QIDS (Quick Inventory of Depressive Symptomatology) total scores. 1A – low inertia (0.03), high innovation (1.52); 1B – high inertia (0.79), low innovation (1.77); 1C – low inertia (0.08), high innovation (4.07); 1D – high inertia (0.66), high innovation (4.51). Values shown for innovation are in standard deviations (s.d.) to aid interpretation.

*Innovation* (*π_j_*), reflects time-specific (level 1) residual variation. Innovation captures the intensity of changes in the data due to unobserved (unmodelled) influences (Hamaker et al., 2018; Jongerling et al., 2015). Using mood as an example, individuals with high innovation display larger differences in the peaks and troughs of their mood over time compared to those with low innovation. When modelling individual differences in innovation it is customary to use the log of the variance, log(*π_j_*), as the dependent variable, to ensure that all values are positive (Hamaker et al., 2018; Parker et al., 2020). Figure 2 shows examples of raw data for individuals with high and low levels of both inertia and innovation in depression scores (QIDS). It is apparent that when individuals with high inertia display increases in depressive symptoms, it takes them longer to return to their average level than those with low inertia, and that individuals with higher innovation report QIDS scores over a broader range of values.

*Cross-lagged effects* (*ϕ_jk_*) refer to the lagged relationships between two variables. In Fig. 1*b*, this is shown as the effect of insomnia at time *t*−1 on depression at time *t* (*ϕ*_DI_, Depression*_t_* regressed on Insomnia*_t_*_−1_,) and in the opposite direction (*ϕ*_ID_, Insomnia*_t_* regressed on Depression*_t_*_−1_). As these cross-lagged effects link the deviations rather than the raw data, they capture the extent to which having worse insomnia (relative to one's individual mean for insomnia) is followed by a *worsening* of mood, and, likewise, the extent to which having poor mood (relative to one's individual mean for mood) is followed by a *worsening* of insomnia.

#### C. Between-person

Cross-lagged panel models are a common tool for analysing longitudinal data for two processes in parallel. An advantage of ILD and the DSEM framework is that we can treat all parameters as *random* so that individuals can take different values. This is illustrated in Fig. 1*c* which shows the between-person model as a set of covariances between these random effects.

#### Analytical steps

We used three analytical steps to address our research questions:

1. Are clinical and demographic characteristics associated with stable and dynamic aspects of sleep and mood?

In Step 1, we estimated unconditional univariate models for each of (hypo)manic, depressive and insomnia symptoms. These models allowed for individual differences in individual mean levels, inertia and innovation. We then incorporated baseline data to examine whether variation in these parameters could be explained by clinical and demographic variables (age, sex, bipolar subtype and history of rapid cycling).

1. Do changes in sleep disturbance predict changes in mood when accounting for their bidirectional interplay?

In Step 2, we estimated unconditional bivariate models between (i) severity of (hypo)manic symptoms and insomnia symptoms, and (ii) severity of depression symptoms and insomnia symptoms. This enabled us to derive correlations between random effects from the two processes (e.g. the correlation between mood inertia and insomnia inertia) and to introduce cross-lagged effects.

1. Do baseline clinical and demographic characteristics moderate these relationships?

In Step 3, by re-estimating each bivariate model across strata defined by the binary clinical and demographic variables, we were able to determine whether the magnitude of the cross-lagged parameters was related to baseline characteristics.

In DSEM, both cross-lagged effects can be modelled simultaneously, thus allowing the magnitude of their estimates to be compared. This is crucial when effects may operate in both directions, as in the present study where effects of mood on later insomnia, and insomnia on later mood, are both plausible and supported by previous studies. When reporting effect sizes for cross-lagged effects we will quote *standardised effects* rather than the raw fixed effect estimates. It is recommended that parameters are standardised *for each person in turn* before taking an average because the magnitude of a cross-lagged effect (*X*→*Y*) for a given person must be interpreted in the context of the variance of both *X* and *Y* (Schuurman, Ferrer, de Boer-Sonnenschein, & Hamaker, 2016). These standardised estimates are called ‘Within-Level Standardised Estimates Averaged Over Clusters’ in Mplus output and ‘average individually standardised’ in Hamaker et al. (2018).

DSEM models are for ‘stable process’ longitudinal data in which scores can fluctuate but there is no systematic change over time (McNeish & Hamaker, 2019). As within-person estimates can be biased by unmodelled systematic change, we used cross-classified time series analysis (Asparouhov et al., 2018) to investigate trends in these data. We found a very modest downward trend in all three measures at the population level, which we accommodated by including linear slopes as an additional random effect in all models. For simplicity, and because the effect of the time trend was not one of our research questions, estimates involving this linear term are not included in the results.

## Results

### Participant characteristics

Among the 649 participants who met inclusion criteria, 400 (61.6%) met DSM-IV criteria for BD-I and 249 met DSM-IV criteria for BD-II (38.4%). Sixty-eight per cent of the sample were women (*N* = 442), and the average age was 52 years (range 22–83). Additional participant characteristics are shown in online Supplementary Table S1.

### Step 1. Univariate models for (hypo)mania, depression and insomnia symptoms

From the unconditional models, the average ‘individual mean levels’ of (hypo)manic, depression and insomnia symptoms around which participants deviated from week to week were 1.98, 7.33, and 3.29, respectively (online Supplementary Table S2). The variance in individual means for (hypo)mania, depression and insomnia was 1.96, 21.10, and 3.71, respectively, indicating substantial heterogeneity in individual means in this population, particularly for depression. There was modest inertia for (hypo)manic (*ϕ*_MM_ = 0.40), depressive (*ϕ*_DD_ = 0.47) and insomnia (*ϕ*_II_ = 0.36) symptoms indicating a tendency for participants’ mood and insomnia to carry over from one week to the next.

Table 1 shows the effect of individual covariates on individual means, inertia and innovation from the univariate models for (hypo)mania (rows 2–5), depression (rows 6–9) and insomnia (rows 10–13). Key results are discussed below.

**Table 1.** Univariate dynamic structural equation models of (hypo)mania, depression and insomnia conditional on baseline covariates

Model	Covariate	Individual mean *μ_j_* (95% CI)	Inertia *ϕ_jj_* (95% CI)	Innovation log(*π_j_*) (95% CI)
(Hypo)mania (ASRM)	BD-II bipolar subtype^a^	0.34 (0.09 to 0.59) *p* = 0.004	0.04 (0.00 to 0.08) *p* = 0.044	0.16 (−0.06 to 0.38) *p* = 0.070
	Female gender^b^	−0.44 (−0.69 to −0.18) *p* < 0.001	−0.03 (−0.07 to 0.02) *p* = 0.131	−0.07 (−0.30 to 0.16) *p* = 0.280
	Older age (⩾55 yrs)^c^	−0.24 (−0.49 to 0.01) *p* = 0.028	−0.01 (−0.05 to 0.04) *p* = 0.370	−0.40 (−0.61 to −0.18) *p* < 0.001
	History of rapid cycling^d^	0.35 (0.05 to 0.64) *p* = 0.009	−0.02 (−0.07 to 0.03) *p* = 0.193	0.66 (0.41 to 0.91) *p* < 0.001
Depression (QIDS)	BD-II bipolar subtype^a^	1.72 (0.96 to 2.49) *p* < 0.001	0.04 (0.00 to 0.08) *p* = 0.015	0.16 (−0.01 to 0.34) *p* = 0.033
	Female gender^b^	0.49 (−0.31 to 1.31) *p* = 0.118	0.02 (−0.02 to 0.06) *p* = 0.139	0.22 (0.03 to 0.40) *p* = 0.011
	Older age (⩾55 yrs)^c^	−1.86 (−2.62 to −1.10) *p* < 0.001	−0.02 (−0.06 to 0.02) *p* = 0.181	−0.49 (−0.66 to −0.32) *p* < 0.001
	History of rapid cycling^d^	1.94 (1.07 to 2.83) *p* < 0.001	−0.01 (−0.06 to 0.04) *p* = 0.326	0.55 (0.35 to 0.74) *p* < 0.001
Insomnia	BD-II bipolar subtype^a^	0.25 (−0.07 to 0.56) *p* = 0.063	0.02 (−0.02 to 0.06) *p* = 0.142	−0.02 (−0.18 to 0.14) *p* = 0.390
	Female gender^b^	0.03 (−0.30 to 0.36) *p* = 0.422	0.00 (−0.04 to 0.04) *p* = 0.472	0.23 (0.07 to 0.39) *p* = 0.003
	Older age (⩾55 yrs)^c^	0.21 (−0.11 to 0.52) *p* = 0.099	−0.02 (−0.06 to 0.01) *p* = 0.089	−0.35 (−0.50 to −0.19) *p* < 0.001
	History of rapid cycling^d^	0.72 (0.35 to 1.07) *p* < 0.001	−0.03 (−0.07 to 0.02) *p* = 0.098	0.38 (0.20 to 0.56) *p* < 0.001

ASRM, Altman Self-Rating Mania Scale; QIDS, Quick Inventory of Depressive Symptomatology; BD-I, Bipolar disorder type 1; BD-II, Bipolar disorder type 2; *p* values quoted are one-tailed as is the convention for MCMC models in Mplus.

Estimates (95% Credible Intervals) in the table indicate the effect of the covariates on individual means, inertia and innovation for each of the three models.

a Reference: BD-I.

b Reference: Male.

c Reference: Age <55 years.

d Reference: No history of rapid cycling.

#### (Hypo)manic symptoms (ASRM)

Participants with BD-II and participants with a history of rapid cycling had higher individual mean levels of (hypo)manic symptoms. However, although BD-II is more common in women, women and older participants had lower individual mean (hypo)mania scores. Participants with BD-II had higher inertia for (hypo)manic symptoms, indicating that when their (hypo)manic symptoms increased from their individual mean levels, these heightened (hypo)manic symptoms persisted longer than those with BD-I. People with a history of rapid cycling had greater innovation, indicating they tended to display larger peaks and troughs in their symptom levels from week to week. Participants who were older had less innovation, indicating reduced variability around individual mean levels.

#### Depressive symptoms (QIDS)

Participants with BD-II and participants with a history of rapid cycling had higher individual mean levels of depression. Older participants had lower individual mean levels. Participants with BD-II had higher depression inertia than those with BD-I. Finally, participants with BD-II, women and those with a history of rapid cycling had greater innovation, whereas older participants had less innovation.

#### Insomnia symptoms

Participants with a history of rapid cycling had higher individual mean levels of insomnia. None of the covariates was associated with insomnia inertia. Women and those with a history of rapid cycling had greater insomnia innovation. In contrast, older participants had lower levels of innovation in insomnia symptoms.

### Step 2. Bivariate models – insomnia and mood

**Fig. 3.**
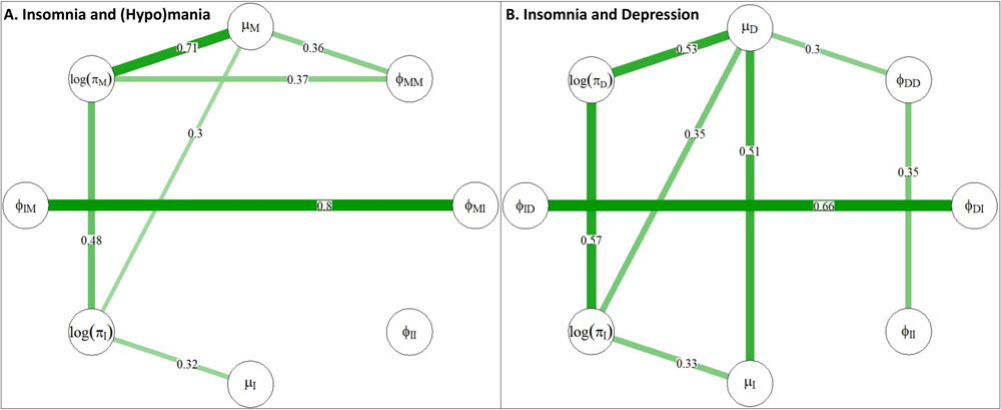
Correlations for between-person effects in bivariate models. Panel A shows bivariate correlations for between-person effects in the Insomnia-(Hypo)mania bivariate model (*μ*_M_ = individual mean levels of (hypo)manic symptoms, *μ*_I_ = individual mean levels of insomnia symptoms, log(*π*_M_) = innovation in (hypo)manic symptoms, log(*π*_I_) = innovation in insomnia symptoms, *ϕ*_MM_ = (hypo)mania inertia, *ϕ*_II_ = insomnia inertia, *ϕ*_MI_ = cross-lagged effect of (hypo)mania at time *t* regressed on insomnia at time *t*−1, *ϕ*_IM_ = cross-lagged effect of insomnia at time *t* regressed on (hypo)mania at time *t*−1). Panel B shows bivariate correlations for between-person effects in the Insomnia-Depression bivariate model (*μ*_D_ = individual mean levels of depression symptoms, *μ*_I_ = individual mean levels of insomnia symptoms, log(*π*_D_) = innovation in depression symptoms, log(*π*_I_) = innovation in insomnia symptoms, *ϕ*_DD_ = depression inertia, *ϕ*_II_ = insomnia inertia, *ϕ*_DI_ = cross-lagged effect of depression at time *t* regressed on insomnia at time *t*−1, *ϕ*_ID_ = cross-lagged effect of insomnia at time *t* regressed on depression at time *t*−1). N.B. The cross-lagged parameters (*ϕ_ij_*) are not average individually standardised for between-person correlations.

The correlations from bivariate models (Fig. 3) describe how groups of individuals who have high values for some parameters also tend to be high on others. Correlations ⩾0.3 between insomnia and mood variables are discussed below.

#### (Hypo)manic and insomnia symptoms

Figure 3*a* shows a strong correlation (0.48) between innovations for insomnia (log(*π*_I_)) and (hypo)mania (log(*π*_M_)), indicating that individuals who had greater variation in week-to-week insomnia symptoms also tended to have greater variation in week-to-week (hypo)mania symptoms. There was a moderate positive correlation (0.30) between individual mean (hypo)mania levels (*μ*_M_) and insomnia innovation (log(*π*_I_)), indicating that individuals with higher typical levels of (hypo)mania experience greater variation in their week-to-week insomnia symptoms. There was a strong correlation (0.80) between cross-lagged effects for insomnia and (hypo)mania (*ϕ*_MI_ and *ϕ*_IM_), indicating that individuals who had stronger effects for prior insomnia on subsequent (hypo)mania were also likely to have a strong effect of prior (hypo)mania on subsequent insomnia.

#### Depression and insomnia symptoms

There was a strong positive correlation (0.51) between individual mean levels of depression (*μ*_D_) and insomnia (*μ*_I_) and a moderate positive correlation (0.35) between individual mean levels in depression (*μ*_D_) and insomnia (log(*π*_I_)) innovations. There was a modest positive correlation between insomnia (*ϕ*_II_) and depression (*ϕ*_DD_) inertia components (0.35). There was also a strong positive correlation (0.57) between depression innovation (log(*π*_D_)) and insomnia innovation (log(*π*_I_)). There was a strong correlation (0.66) between cross-lagged effects for insomnia and depression (*ϕ*_DI_ and *ϕ*_ID_), indicating that individuals who had stronger effects for prior insomnia on subsequent depression were also likely to have a strong effect of prior depression on subsequent insomnia.

### Step 3. Bivariate models – stratification by baseline characteristics

Finally, we describe the cross-lagged relationship between insomnia and mood. As described above, we derive ‘average individually standardised’ cross-lagged effects – estimates which are standardised for each participant before averaging across all participants (Schuurman et al., 2016). With traditional regression models, the cross-lagged estimates would be a pair of constants which apply to all members of the population. Here we use random effects which permit the magnitude of the cross-lagged relationships to vary between individuals.

#### (Hypo)manic and insomnia symptoms

The average cross-lagged effect from insomnia to (hypo)mania was 0.007 [95% confidence interval (CI) −0.003 to 0.015, *p* = 0.106] with the corresponding effect from (hypo)mania to insomnia being 0.013 (95% CI 0.001 to 0.023, *p* = 0.014). The modest evidence for effects between insomnia and (hypo)mania disguise strong subgroup differences, shown in Table 2. We see positive cross-lagged effects between (hypo)mania and insomnia for individuals with BD-I and, in contrast, weaker negative effects for those with BD-II. The magnitude of the cross-lagged effects between (hypo)mania and insomnia varied by gender (stronger for females) and the sign of the effect of (hypo)mania on subsequent insomnia was negative for those with a history of rapid cycling.

**Table 2.** Differences in the magnitude of the average individually standardised cross-lagged effects with respect to baseline covariates

Covariate		(Hypo)mania (ASRM) and Insomnia		Depression (QIDS) and Insomnia	
		(Hypo)mania*_t_*_−1_→Insomnia*_t_*	Insomnia*_t_*_−1_→(Hypo)mania*_t_*	Depression*_t_*_−1_→Insomnia*_t_*	Insomnia*_t_*_−1_→Depression*_t_*
Bipolar subtype	BD-I	0.031 (0.007)	0.015 (0.006)	0.051 (0.007)	0.026 (0.006)
	BD-II	−0.013 (0.008)	−0.004 (0.008)	0.091 (0.009)	0.013 (0.007)
	Difference	−0.044 (−0.065 to −0.023) *p* < 0.001	−0.019 (−0.039 to 0.001) *p* = 0.058	0.040 (0.018 to 0.062) *p* < 0.001	−0.013 (−0.031 to 0.005) *p* = 0.159
Gender	Male	0.000 (0.009)	−0.010 (0.009)	0.056 (0.009)	0.022 (0.008)
	Female	0.019 (0.006)	0.016 (0.006)	0.069 (0.007)	0.021 (0.006)
	Difference	0.019 (−0.002 to 0.040) *p* = 0.079	0.026 (0.005 to 0.047) *p* = 0.017	0.013 (−0.009 to 0.035) *p* = 0.255	−0.001 (−0.021 to 0.019) *p* = 0.920
Age	<55 years	0.013 (0.007)	0.010 (0.008)	0.069 (0.007)	0.035 (0.008)
	⩾55 years	0.009 (0.008)	0.009 (0.008)	0.057 (0.009)	0.000 (0.008)
	Difference	−0.004 (−0.025 to 0.017) *p* = 0.707	−0.001 (−0.023 to 0.021) *p* = 0.930	−0.012 (−0.034 to 0.010) *p* = 0.293	−0.035 (−0.057 to −0.013) *p* = 0.002
History of rapid cycling	No	0.017 (0.006)	0.002 (0.005)	0.067 (0.006)	0.024 (0.006)
	Yes	−0.013 (0.011)	0.018 (0.011)	0.063 (0.012)	0.020 (0.010)
	Difference	−0.030 (−0.055 to −0.005) *p* = 0.017	0.016 (−0.008 to 0.040) *p* = 0.186	−0.004 (−0.030 to 0.022) *p* = 0.766	−0.004 (−0.027 to 0.019) *p* = 0.732

Values in parentheses denote standard errors, results in square brackets denote 95% CIs. *p* values quoted are two-tailed.

#### Depression and insomnia symptoms

The average cross-lagged effect from insomnia to depression was 0.022 (95% CI 0.014 to 0.030, *p* < 0.001) and from depression to insomnia 0.065 (95% CI 0.054 to 0.075), *p* < 0.001). We found evidence of subgroup differences for age and bipolar subtype – an effect of insomnia on subsequent depression was observed among younger participants, and there was evidence of a greater effect of depression on subsequent insomnia for those with BD-II.

## Discussion

Using ILD, we modelled stable and dynamic relationships between sleep and mood in a large sample of individuals with BD. Novel statistical methods allowed us to derive indices of sleep and mood stability/variability that align with features of BD that will be familiar to clinicians and those with lived experience of the condition. It is well-known that some patients will experience more variable mood symptoms, including insomnia; here we have quantitatively derived estimates of these clinical features from prospective mood rating data. We also disaggregated within- and between-person heterogeneity and explored the bidirectional relationship between sleep and mood. Furthermore, we were able to examine the extent to which bidirectional effects were moderated by clinical and demographic characteristics.

We found that BD subtype, gender, a history of rapid cycling and age were associated with dynamic and stable characteristics of (hypo)mania, depression and insomnia. These associations differed depending on which dynamic or stable aspect was examined. For example, despite being conceptualised often as a less severe form of bipolar illness, people with BD-II experienced higher average levels of depression and (hypo)mania, and greater peaks and troughs in how their depressive symptoms fluctuated over time. Once they experienced changes in depression or (hypo)manic symptoms, people with BD-II also took longer to return to their usual mood levels. These findings are consistent with previous longitudinal research finding that people with BD-II, despite experiencing less severe manic episodes, have a severe course of illness characterised by greater mood instability (Faurholt-Jepsen et al., 2015; Lamers et al., 2018; Szmulewicz, Martino, & Strejilevich, 2019) and a greater proportion of time with depressive and hypomanic symptoms (Judd et al., 2003) than those with BD-I. These findings are relevant for clinicians particularly since BD-II is generally considered to be a less severe form of the disorder, and therefore the fact that patients with BD-II can be more impacted by their mood disorder day-to-day than those with BD-I may seem counter-intuitive. Our results also indicate that patients who are young, have a history of rapid cycling and are women could be more likely to show mood instability and erratic sleep, and taking account of these factors may be important in identifying those at greatest risk of these problems. In addition, those with more variable sleep had worse mood over the course of the study, indicating that patients whose sleep varies more from one week to the next may be at higher risk of chronic mood problems. Future research should investigate whether sleep interventions impact on mood symptoms more generally.

We investigated whether changes in sleep predicted changes in mood. Prior research suggests that this relationship is bidirectional (Buysse et al., 1999; 2008; Combs et al., 2014; Hertenstein et al., 2019; Kahn, Sheppes, & Sadeh, 2013; Kalmbach, Pillai, Roth, & Drake, 2014), however here we extend this by modelling the relationship between sleep and mood in both directions simultaneously. We found that, on average, insomnia is an early indicator of worsening depressive symptoms independent of mood state at the time insomnia becomes apparent, and independent of other ways in which sleep and mood vary over time. In contrast and perhaps unexpectedly, on average, increases in insomnia symptoms did not predict subsequent increases in manic symptoms. However, we observed substantial variation between participants, suggesting that there may be a subgroup where insomnia is a useful predictor. This concords with findings from a previous study of 59 individuals with BD, which found that reduced sleep duration predicted increases in next day manic symptoms in only 34% of participants (Bauer et al., 2006).

Prior studies have found evidence of individual differences in the prospective relationship between sleep and mood in bipolar populations (Bauer et al., 2006; Colombo, Benedetti, Barbini, Campori, & Smeraldi, 1999; Wu & Bunney, 1990). The DSEM approach allowed us to model these individual differences and investigate whether clinical and demographic characteristics explain them. Specifically, we investigated BD subtype, gender, age, and rapid cycling as potential moderators of the magnitude of the sleep-mood relationship. We found that the strength of the bidirectional associations between sleep and mood depended on several characteristics. We found stronger sleep-(hypo)mania relationships in those with BD-I and stronger depression-insomnia relationships in BD-II. We also found strong evidence for moderation by gender (stronger sleep-(hypo)mania relationships for females) and by age (stronger sleep-depression relationships for younger people). This has implications for studies examining the relationship between sleep and mood in BD, as the strength of association will depend on the characteristics of the sample. This could explain why the results from studies conducted in subgroups (e.g. rapid cycling, Leibenluft et al., 1996) are not always replicated in other samples. The fact that not all patients with BD will experience mood changes following sleep loss, and that this may be influenced by bipolar subtype, gender and age, is of clinical relevance since it raises the possibility of more personalised treatment approaches, specifically targeting sleep interventions based on patient characteristics, but again this requires further work. Future research should explore whether these findings replicate using daily data, and when examining mood episodes in addition to symptoms.

### Strengths and limitations

We had access to a large sample of people with BD who provided data at frequent intervals over a long duration. Such data allowed us to investigate novel questions about the sleep-mood relationship in BD, to tease apart the bidirectional relationship between sleep and mood, and examine individual differences in this relationship. However, our results should be considered in light of limitations stemming from the fact that the True Colours system was initially set up for clinicians to assess the patterns of symptoms in individual patients and use as a reference during consultations.

The ASRM is primarily designed to detect the presence of (hypo)mania in a single patient rather than for use in population-based studies. Whilst the ASRM may be an ideal measure clinically, it might have limited sensitivity for capturing change over time. A drawback of our statistical methods was that we had to exclude participants with no variation in responses, usually due to their ASRM data, as variation in depressive symptoms was more common (see online Supplement). The symptoms of (hypo)mania that fluctuate more readily over time may be of less interest clinically, and whilst it is tempting to propose that the recently developed longer version of the ASRM (Altman & Østergaard, 2019) might be administered in future studies, longer scales mean greater participant burden and more drop-out, particularly with intensive data collection.

We did not have information on medication use, which could have influenced our results. We also do not know whether medications were used prophylactically or in response to participants experiencing changes in sleep, which may explain the weak bidirectional relationship between insomnia and (hypo)mania. Moreover, the sample may not be representative of individuals with BD, as it comprised participants who were motivated to complete weekly questionnaires for an extended period. The sample was over-representative of older individuals, who had lower severity of mood symptoms over time and were of high socioeconomic status (i.e. higher education, professional occupation). Investigation of whether these results are replicated in more representative, clinical samples with medication information is needed. Insomnia symptoms were also the only sleep phenotype available in this study. These were items from the QIDS and therefore may have limited utility for capturing sleep compared to more comprehensive sleep questionnaires and methods such as actigraphy. The insomnia items also assessed the perception of insufficient sleep rather than total hours slept. This may explain why we find weaker evidence for longitudinal associations between insomnia and (hypo)mania, as this does not capture the reduced need for sleep, a potentially crucial measure in this scenario. Future research should explore prospective relationships between sleep and mood using a broader range of sleep and circadian phenotypes.

Finally, in the current study, these estimates are specific to the one-week lag afforded by these data and some processes may be operating more strongly on a longer/shorter timescale (Deboeck & Preacher, 2015; Gollob & Reichardt, 1987). For example, Bauer et al. (2006) demonstrated mood changes within days of sleep changes. Future research applying DSEM to daily data (and data collected within a day) will be important for further understanding the relationship between sleep and mood in this population.

## Conclusion

In conclusion, using ILD and novel statistical methods, we were able to examine stable and dynamic relationships between sleep and mood in individuals with BD. We found that increased variability in insomnia symptoms was associated with increased mood variability and worse mood over the course of the study. There was strong evidence of bidirectional relationships between insomnia and depressive symptoms but only weak support for bidirectional relationships between insomnia and (hypo)manic symptoms. However, there was substantial variability between participants in the strength of prospective associations between insomnia and mood, which depended on age, gender, bipolar subtype, and a history of rapid cycling. Our results highlight the importance of monitoring sleep in people with BD and considering subgroup differences in the sleep-mood relationship. As advances in digital technology increase the availability of ILD on sleep and mood, novel methods to analyse these data present an exciting opportunity for furthering our understanding of BD and other mood disorders, and for translating that understanding towards personalised interventions.

## Supplementary material

For supplementary material accompanying this paper visit https://doi.org/10.1017/S0033291721005377. click here to view supplementary material
