# Supplementary material for: The dynamic interplay between sleep and mood: an intensive longitudinal study of individuals with bipolar disorder
Source: Psychol Med. 2022 Jan 25;53(8):3345–54. doi: 10.1017/S0033291721005377 (PMC10277721; doi:10.1017/S0033291721005377)
Supplement: Supplementary file 1 [file S0033291721005377sup001.docx]

Supplement

# Sample selection

True Colours data were downloaded on the 25 September 2019, at which time 925 BDRN participants were enrolled in True Colours and met DSM-IV criteria for BD-I or BD-II. Inclusion criteria for our analyses were as follows. First, we selected all participants who had enrolled on or before 25 December 2017 and selected the first 21 months of data for each participant. This was to ensure that all participants had the same opportunity to provide data, while maximizing the number of participants and the length of time participants were enrolled in the system. These constraints were chosen empirically and aimed to balance the competing needs of ensuring both a large sample for analysis and a long period of follow-up. This reduced the sample to 882. Second, it was necessary to exclude individuals with no variation in their data as this can cause estimation problems (1,2). Therefore, participants with no variability in either mania, depression or insomnia symptoms were excluded. Of these, 31 participants had no variability in their data because they had only responded on one occasion during the 21-month period. Of the participants who had responded more than once, 6 had no variation in QIDS scores, 42 had no variation in ASRM scores, and 18 had no variation in insomnia scores. In line with current guidance, participants with few datapoints (i.e., less than 3 months of data in total) and large amounts of missing data in between responses (i.e., a gap of more than 6 months) were also excluded (n=149). This resulted in a sample of 649 participants.

## References

1. Asparouhov T, Hamaker EL, Muthén B. Dynamic Structural Equation Models. Struct Equ Model A Multidiscip J [Internet]. 2018 May 4;25(3):359–88. Available from: https://doi.org/10.1080/10705511.2017.1406803

2. Dzubur E, Ponnada A, Nordgren R, Yang C-H, Intille S, Dunton G, et al. MixWILD: A program for examining the effects of variance and slope of time-varying variables in intensive longitudinal data. Behav Res Methods [Internet]. 2020 Aug 2;52(4):1403–27. Available from: http://link.springer.com/10.3758/s13428-019-01322-1

**Table S1.** Demographic and Clinical Characteristics of Participants (N = 649)

| Variable |  |
| --- | --- |
| DSM-IV Diagnosis |  |
| BD-I, *n* (%) | 400 (61.6) |
| BD-II, *n* (%) | 249 (38.4) |
| Missing, *n* (%) | 0 (0.0) |
| Gender |  |
| Male, *n* (%) | 207 (31.9) |
| Female, *n* (%) | 442 (68.1) |
| Missing, *n* (%) | 0 (0.0) |
| Age (years) |  |
| < 55 years, *n* (%) | 383 (59.0) |
| ≥ 55 years, *n* (%) | 266 (41.0) |
| Missing, *n* (%) | 0 (0.0) |
| History of Rapid Cycling |  |
| No, *n* (%) | 480 (74.0) |
| Yes, *n* (%) | 151 (23.3) |
| Missing, *n* (%) | 18 (2.8) |
| Education |  |
| No Higher Education, *n* (%) | 232 (35.7) |
| Higher Education, *n* (%) | 327 (50.4) |
| Missing, *n* (%) | 90 (13.9) |
| Highest Occupation |  |
| Never worked, *n* (%) | 10 (1.5) |
| Non-professional, *n* (%) | 206 (31.7) |
| Professional, *n* (%) | 352 (54.2) |
| Missing, *n* (%) | 81 (12.5) |
| True Colours Variables |  |
| Mania (ASRM) score, mean (SD) | 2.0 (1.8) |
| Depression (QIDS) score, mean (SD) | 7.1 (4.7) |
| Insomnia score, mean (SD) | 3.2 (1.9) |
| Number of responses, median (IQR) | 74 (46) |
|  |  |

BD-I, bipolar disorder type 1; BD-II, bipolar disorder type 2; ASRM, Altman Self-Rating Mania Scale; QIDS, Quick Inventory of Depressive Symptomatology. ^1^Mean scores for True Colours data were calculated for each individual and then averaged across the whole sample. ^2^Sleep items have been removed from the ASRM and QIDS total scores, this does not affect the QIDS score range but does limit the ASRM total score range to 0-15.

# Univariate Models: Correlations for all between-person relationships

**Table S2.**  Means, variances and correlations for the three key components from each univariate model.

|  | **Mania (ASRM)** | | |  | **Depression (QIDS)** | | |  | **Insomnia** | | |
| --- | --- | --- | --- | --- | --- | --- | --- | --- | --- | --- | --- |
|  | **Individual** **mean** μ_M_ | **Inertia** φ_MM_ | **Innovation** log(π_M_) |  | **Individual** **mean** μ_D_ | **Inertia** φ_DD_ | **Innovation** log(π_D_) |  | **Individual** **mean** μ_I_ | **Inertia** φ_II_ | **Innovation** log(π_I_) |
|  |  |  |  |  |  |  |  |  |  |  |  |
| **Mean** | 1.98 (1.85, 2.12) | 0.40 (0.38, 0.42) | 0.68 (0.58, 0.79) |  | 7.33 (6.94, 7.72) | 0.47 (0.45, 0.49) | 1.91 (1.82, 1.99) |  | 3.29 (3.13, 3.44) | 0.36 (0.34, 0.38) | 0.31 (0.23, 0.39) |
| **Variance** | 1.96 (1.62, 2.32) | 0.06 (0.05, 0.07) | 1.87 (1.66, 2.10) |  | 21.10 (18.61, 23.92) | 0.05 (0.04, 0.06) | 1.13 (1.01, 1.27) |  | 3.71 (3.30, 4.18) | 0.04 (0.03, 0.04) | 0.96 (0.85, 1.08) |
|  |  |  |  |  |  |  |  |  |  |  |  |
| **Individual** **mean** | 1.00 |  |  |  | 1.00 |  |  |  | 1.00 |  |  |
| **Inertia** | 0.37 | 1.00 |  |  | 0.32 | 1.00 |  |  | 0.18 | 1.00 |  |
| **Innovation** | 0.71 | 0.37 | 1.00 |  | 0.53 | 0.20 | 1.00 |  | 0.33 | 0.12 | 1.00 |
|  |  |  |  |  |  |  |  |  |  |  |  |

Values in parentheses are 95% Credible Intervals. ASRM, Altman Self-Rating Mania Scale; QIDS, Quick Inventory of Depressive Symptomatology.
